# The Control of Infantile Mortality

**Published:** 1908-05-16

**Authors:** 


### Public Health and Hygiene.

### THE CONTROL OF INFANTILE MORTALITY.

In our issue of November 30 we drew attention to the influence of the cold summer of 1907 upon the death-rate, and exhibited by means of diagrams and tables the fact that the low infantile mortality of the third quarter of the year was mainly responsible for the diminished death-rate for this quarter in the 76 great towns. We further pointed out that " the prevailing type of weather had accomplished what for several years it had been the aim of Medical Officers of Health and others engaged in public-health work to bring about—namely, a marked reduction in the deaths from infantile diarrhœa "—and we inferred this to be due to the absence of those causal meteorological conditions on which the prevalence of this disease is dependent.

There has recently been circulated a report by the Medical Officer of Health of Huddersfield in which the rapid and phenomenal decline in the infantile mortality rate of that town was shown on a chart, and an attempt made to relate this decline to the excellent and well-known efforts made in Huddersfield to bring about this result.

The Medical Officer of Health of Bristol has just issued a report in which, side by side with the Huddersfield chart, is set out a corresponding chart of the infantile mortality in Bristol over an identical period. The resemblance during recent years of Huddersfield and Bristol in respect of infantile mortality is very striking. Dr. Davies carries the comparison further by placing alongside the Huddersfield report a corresponding report for Bristol.

Huddersfield Infant Mortality.

| — | Births Registered | Deaths under 1 year of age | Infant Mortality Figure | — |
|---|---|---|---|---|
| **1907.** | 2,189 | 212 | 97 | Reduction 28 per cent. |
| Preceding 10 years—1897-1906 ... ... | 22,991 | 3,104 | 135 | — |
| The 3 years during which special work against infant mortality has been in progress, 1905-6-7 | 6,746 | 792 | 117 | Reduction 18 per cent. |
| Preceding 10 years—1895-1904 ... ... | 22,631 | 3,215 | 142 | — |

Estimated population, 1907 ... ... 94,814

Bristol Infant Mortality.

| — | Births Registered | Deaths under 1 year of age | Infant Mortality Figure | — |
|---|---|---|---|---|
| **1907.** | 8,915 | 900 | 100.9 | Reduction 26 per cent. |
| Preceding 10 years—1897-1906 ... ... | 89,535 | 12,151 | 135 | — |
| The 3 years, 1905-6-7 ... | 27,936 | 3,278 | 117.3 | Reduction 15 per cent. |
| Preceding 10 years—1895-1904 ... ... | 83,673 | 11,616 | 138.7 | — |

Estimated population, 1907 ... ... 367,979

Referring to these comparative reports, Dr. Davies says: " It is at once evident that the wholesale improvement in the Huddersfield infant mortality returns, claimed as due to the special measures taken there, are not proved by the figures adduced to be due either entirely or in any large degree to these measures; for a practically identical improvement has taken place during the same years in Bristol without any special methods to this end having been taken."

While fully in sympathy with the measures being taken to reduce infantile mortality, and ready to admit that the slight difference between the Huddersfield and Bristol figures may be due to the special measures adopted in Huddersfield, Dr. Davies insists on the need for care in the " use of statistical methods, lest too much be claimed and disappointment result."

